# Developing a system for in vivo imaging of maize roots containing iodinated contrast media in soil using synchrotron XCT and XRF

**DOI:** 10.1007/s11104-020-04784-x

**Published:** 2020-12-10

**Authors:** Callum P. Scotson, Arjen van Veelen, Katherine A. Williams, Nicolai Koebernick, Dan McKay Fletcher, Tiina Roose

**Affiliations:** 1 Bioengineering Sciences Research Group, Department of Mechanical Engineering, School of Engineering, Faculty of Engineering and Physical Sciences, University of Southampton, University Road, Southampton, SO17 1BJ UK; 2 Material Science and Technology Division, Los Alamos National Laboratory, Los Alamos, NM 87545 USA; 3 Stanford Synchrotron Radiation Lightsource, SLAC National Accelerator Laboratory, Menlo Park, CA 94025 USA; 4 Soil Science and Soil Protection, Martin Luther University Halle-Wittenberg, Von-Seckendorff-Platz 3, 06120 Halle (Saale), Germany

**Keywords:** Roots, Maize, Soil, X-ray computed tomography, Iodinated contrast media, X-ray fluorescence mapping

## Abstract

**Aims:**

We sought to develop a novel experimental system which enabled application of iodinated contrast media to in vivo plant roots intact in soil and was compatible with time-resolved synchrotron X-ray computed tomography imaging. The system was developed to overcome issues of low contrast to noise within X-ray computed tomography images of plant roots and soil environments, the latter of which can complicate image processing and result in the loss of anatomical information.

**Methods:**

To demonstrate the efficacy of the system we employ the novel use of both synchrotron X-ray computed tomography and synchrotron X-ray fluorescence mapping to capture the translocation of the contrast media through root vasculature into the leaves.

**Results:**

With the application of contrast media we identify fluid flow in root vasculature and visualise anatomical features, which are otherwise often only observable in ex vivo microscopy, including: the xylem, metaxylem, pith, fibres in aerenchyma and leaf venation. We are also able to observe interactions between aerenchyma cross sectional area and solute transport in the root vasculature with depth.

**Conclusions:**

Our novel system was capable of successfully delivering sufficient contrast media into root and leaf tissues such that anatomical features could be visualised and internal fluid transport observed. We propose that our system could be used in future to study internal plant transport mechanisms and parameterise models for fluid flow in plants.

**Supplementary Information:**

The online version contains supplementary material available at 10.1007/s11104-020-04784-x.

## Introduction

X-ray computed tomography (XCT) is a commonly employed technique for biomedical imaging research (Kalender 2006), and it is increasingly used for non-destructive 3D imaging of plant root and soil systems (Mooney et al. 2012; Roose et al. 2016). However, due to overlapping X-ray attenuation of soil pore water and root tissues, the ability to distinguish between soil and plant roots remains a challenge (Mooney et al. 2012) – the resulting poor contrast complicates image segmentation and subsequent quantifications. Additionally, higher X-ray energies are required to penetrate larger samples and these higher energies are proportionally less sensitive to attenuation. Therefore the maximum attainable contrast-to-noise ratio generally decreases as sample diameter increases (Attix 2008) and thus contrast issues can also limit sample sizes.

When similar issues occur within biomedical soft tissue imaging, it is common practice to use solutions containing radiopaque elements as contrast agents – iodine in particular (Lusic and Grinstaff 2013). More specifically, in biomedical imaging non-ionic iodinated contrast media are commonly used since they possess several favourable traits (Lusic and Grinstaff 2013). Firstly, they are generally less reactive compared to alternative contrast media – for example, ionic iodinated contrast media (Aspelin 2006; Lusic and Grinstaff 2013). Non-ionic iodinated contrast media are also highly soluble and possess a viscosity similar to water (Lusic and Grinstaff 2013). This is advantageous when the contrast media is to be used in organic tissues and particularly when the intention is to utilise the contrast media in time-resolved studies where excessive toxic effects resulting from the reactivity of the contrast media could damage tissues over time and thus alter tissue structures.

There are currently very few examples of the use of contrast agents within in vivo plant material; most existing studies have employed staining of harvested material (Ahn et al. 2010; Blonder et al. 2012; Dhondt et al. 2010). However, when imaging ex vivo or harvested plant material it is not possible to capture plant processes over time and much structural information can be lost – particularly when studying root systems which are removed from soil. Karunakaran et al. (2015) and Keyes et al.*,* (2017b) are two of only very few examples of the use of contrast media for in vivo XCT phytological imaging. Karunakaran et al. (2015) used the non-ionic iodinated contrast agent Ioversol to undertake synchrotron phase contrast imaging of wheat seed spikes and canola stems by introducing the contrast media directly into the vasculature via injection. The resulting images effectively captured the internal structure of the wheat seed spikes and canola stems and indicated the potential for using iodinated contrast medium as an effective flow tracer in in vivo phytological systems. The study suggested that translocation via plant vasculature is a promising avenue to deliver contrast media to different plant tissues for synchrotron X-ray computed tomography (SRXCT) imaging. However, the plant samples featured in this study were imaged against a background of air which is considerably less attenuating than soil and thus the complications of limited contrast introduced when imaging plant material in soil were not present. Keyes et al., (2017b) submerged cut leaf material of a live winter pea plant in non-ionic iodinated contrast media (both Gastrografin and Niopam contrast agents) and after 24 h undertook XCT imaging of the in vivo soil borne roots to capture contrast media which had translocated from the leaves to the roots via the plant’s vasculature. This further confirmed the promising potential for using iodinated contrast media to image in vivo root systems. However, the difficulty of this experimental setup is that it is complex to estimate the time required for the contrast media to translocate through the plant to the root system and also that it could be difficult to know how much contrast media is likely to reach the root system in plants of various size and scale.

Alternative approaches for capturing root anatomical information or for tracing fluid flow in root-soil systems have limitations with regard to the resolution and dimensionality of the data which can be collected. Whilst microscopy imaging techniques, such as SEM, can provide substantial detail at high magnification and resolution, this technique is generally limited to capturing ex vivo tissues at a single time-point in 2D (Hall and Hawes 2012). Therefore, such a technique cannot practicably trace spatial fluid flow through in vivo root systems intact within soil over time. Chemical sampling techniques, such as suction cups or microdialysis, can be used to capture time-resolved fluid flow through plant and soil systems. However, these approaches can only provide a coarse spatial resolution (Fletcher et al. 2019; Gottlein et al. 1996; Miro and Frenzel 2004; Petroselli et al. 2020; Vetterlein and Jahn 2004). In using XCT it is possible to capture 3D spatial information for time-resolved fluid flow and transport of iodinated contrast media in live plant roots intact within soil. In particular, by using SRXCT it is possible to achieve fast data acquisition, a high signal to noise ratio and to utilise phase contrast. Although laboratory-based XCT analyses can capture 3D information of internal plant structure in situ and in vivo*,* the data collection is generally too slow to acquire high resolution information of iodinated media passing through root and soil systems prone to movement. Whilst iodine is not an essential plant nutrient, typical plant nutrients are not radiopaque and thus cannot be visualised using SRXCT. Therefore, iodine can be used as an SRXCT-visible analogue for solutes which might typically be taken up plants. The ability to capture spatiotemporal fluid flow within live roots intact in soil is of importance for improving our understanding of internal solute transport mechanisms and physiological features, such as internal root structure, which may influence this transport.

The aim of our study was to develop an experimental system which enables the contrast media to be applied directly to an exposed section of root for uptake and translocation, and which is also compatible with time-resolved SRXCT to facilitate in vivo imaging of plant root material in soil. We hypothesised that by supplying the contrast media directly to the roots we would circumnavigate the issues of translocation highlighted above in the work of Karunakaran et al. (2015) and Keyes et al., (2017b). In this study we demonstrate the efficacy of this experimental setup and outline example data which can be gathered using such a system. For example, we study the influence of aerenchyma cross sectional area on solute translocation through the cortex – aerenchyma is a plant tissue which contains enlarged gas spaces, often an indicator of environmental stresses such as hypoxia, and has previously been observed to impede axial transport through the root cortex (Evans 2004; Fan et al. 2007; Hu et al. 2014). We explored the SRXCT imaging of maize roots which had been partially submerged in iodinated contrast media whilst the remainder of the root material remained in soil. From this data we were able to observe the translocation of the iodinated contrast media through the root cortex to the stele and into above ground tissues such as the leaves and stem. We also used synchrotron X-ray fluorescence (SRXRF) to confirm the presence of iodine in above ground plant tissues.

## Materials and methods

### Experimental system

#### Soil imaging experimental system assembly

**Fig. 1:**
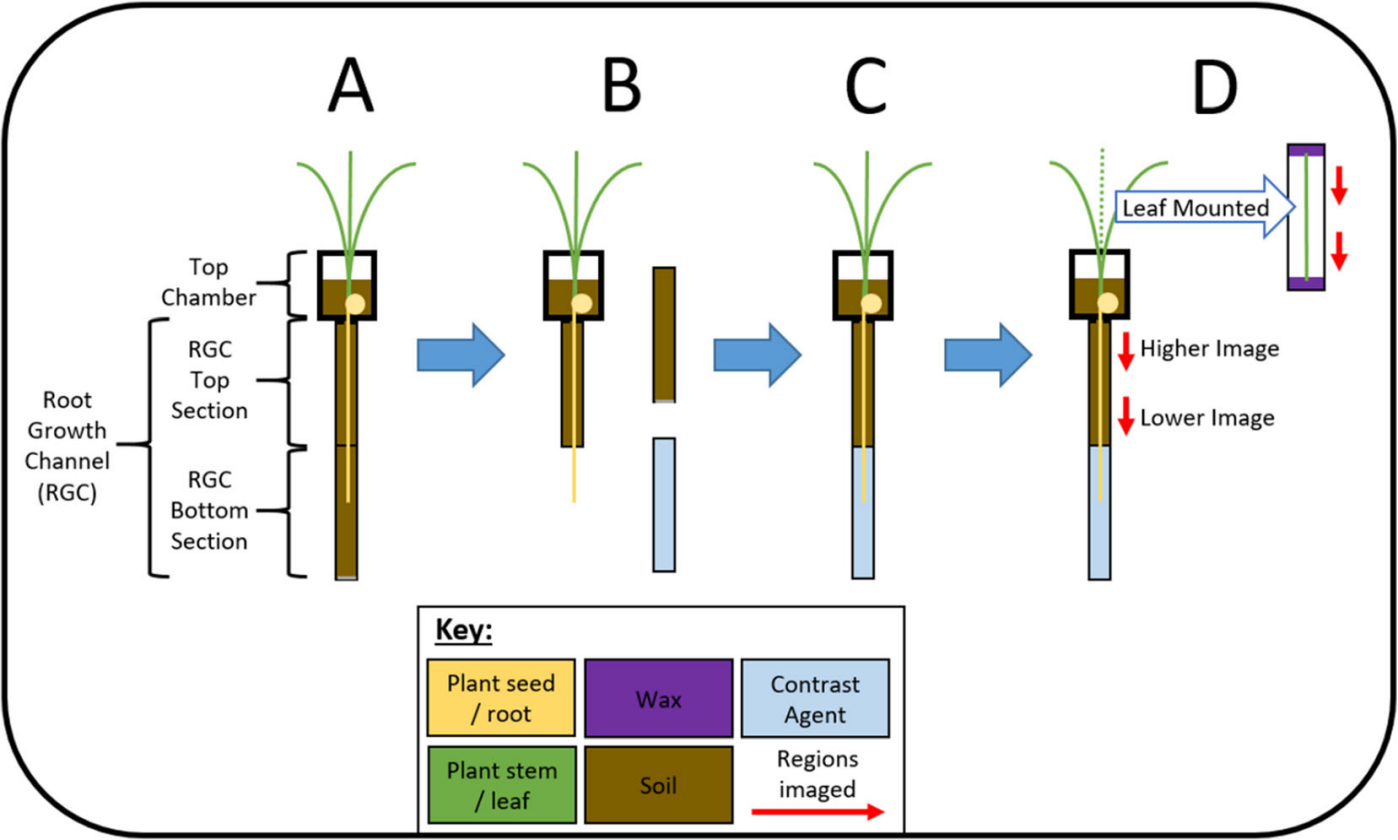
Schematic of the experimental systems used for growth and imaging of the maize plants. This displays all four stages of the experimental setup including: the initial system setup for plant growth and root development (A), the removal of the basal section of the root chamber (B), the replacement of the basal section of the root chamber with the contrast agent (C) and the final setup displaying the various regions which underwent SRXCT imaging of both the roots and excised leaf material (D). Red arrows indicate the regions of the roots and leaves which were imaged using SRXCT. A colour key is placed at the bottom of the schematic indicating the colour of plant roots, plant leaves, soil, iopamidol contrast media and the SRXCT imaged region

The experimental system used for the imaging of roots in soil consisted of two main components: a 3D printed top chamber and, connected to the base of this top chamber, a root growth channel constructed from 1 mL syringe barrels (Fig. 1). This system was setup to enable in vivo SRXCT imaging of roots in the basal root growth channel and was designed for ease of exposure of the root tip to the contrast media by removal of a section of soil after root growth. The 3D printed top chamber was a cylinder of 2 cm in diameter and 3 cm in depth. The top end of this cylinder was open but the basal end was closed except for a 3 mm hole in the centre over which the root growth channel would be placed. The root growth channel was composed of two 4 cm lengths of 1 mL syringe barrels (BD Plastipak, NJ, USA) with an internal diameter of 4.88 mm. The two lengths of syringe barrel were joined end to end by waterproof insulation tape. The exposed end of the top section of root growth channel was attached to the base of the top chamber over the basal hole (Fig. 1a). The exposed end of the basal section of the root growth channel was covered with a single layer of nylon mesh with an aperture of 300 μm (Plastok, UK) such that water could enter the syringe barrel, but larger soil particles would not be able to leave. Three such systems were assembled to provide three replicates and these were referred to as ‘Maize 1’, ‘Maize 2’ and ‘Maize 3’ or in shorter form as M1, M2 and M3.

#### Plant growth conditions

Maize seeds (*Zea mays* L. cv. tasty sweet trophy F1) were germinated and then planted in the top chamber of the system. To germinate these seeds, they were placed onto damped laboratory blue roll in a petri dish for a period of 10 days at 25 °C and covered from light. After this ten day period the seedling root had emerged and was approximately 1 cm in length. The seedling was placed into the top chamber of the experimental system and the seedling root was manoeuvred through the basal hole in the top chamber into the top of the root growth channel. A sand-textured Eutric Cambisol field soil (collected from a surface plot at Abergwyngregyn, North Wales, UK: 53.0140 N, −4.0010 W), sieved to 2 mm and below, was then poured into the top chamber and tapped down gently until it filled the root growth chamber and covered the seedling in the top chamber.

To facilitate growth of the maize seedlings in the experimental systems, the systems were placed into a Conviron Growth Cabinet for a period of ten days prior to the imaging experiments. Ten days was sufficient to ensure that the roots had grown through the top root chamber and into the bottom root chamber section without being too long a length of time such that the roots would begin to grow too large for the system. The temperature in the cabinet was maintained at a temperature of 25 °C and humidity of 60%. The cabinet had a ‘daylight’ period of 14 h and a ‘night’ period of 10 h. The seedlings were watered every day by pipetting 1 mL of water onto the soil in the top chamber and the basal end of the root chamber, covered by the nylon mesh, was placed into a tray of water to a depth of 1 cm.

#### Addition of contrast media

Following the capture of the ‘Control’ images, contrast media was added. First, the bottom section of the root growth channel was detached from the top section of the root growth chamber (Fig. 1b), leaving a length of root exposed but undamaged. The removed section was then replaced with an empty section which was sealed watertight at one end and filled with iopamidol iodinated contrast media (trade name: Niopam) at a concentration of 185 mg iodine / ml (Fig. 1c) - the roots extending from the base were thus submerged in the contrast media. This concentration was determined by previous work (Scotson et al. 2020) and was confirmed as appropriate with the use of a phantom containing the contrast media and soil which displayed sufficient contrast between materials. The phantom consisted of a length of syringe identical to those used for the root growth chamber. This length of syringe was sealed at one end, filled with soil and then the soil was saturated with contrast media at the concentration of 185 mg iodine / ml. An SRXCT image of the phantom was then captured and the concentration was deemed adequate when a discrete grey value peak was produced by the contrast media within grey value histograms.

### Synchrotron X-ray CT imaging

The SRXCT imaging was undertaken at three time-points: once before the addition of contrast media (‘Control’), 11 h after the addition of the contrast media (‘T1’) and 23 h after the addition of the contrast media (‘T2’). The plants were not imaged more frequently than this owing to concerns over the effects of prolonged X-ray exposure on plant health. Zappala et al. (2013) previously observed that plant health was not significantly affected provided plants were not exposed to X-rays for prolonged periods. This has also been confirmed by Keyes et al., (2017a) who monitored microscale plant root growth using SRXCT and were able to mitigate the negative effects of X-rays on plant health by limiting exposure. Finally, sections of leaf and stem, excised and mounted as described below, were imaged 25 h after the initial addition of contrast media (‘Leaf Images’). The synchrotron XCT imaging was carried out at the I13–1 beamline at Diamond Light Source (DLS), Didcot, UK. The energies used for the SRXCT was 15–20 keV (‘pink beam’). A total of 1601 equiangular projections were recorded through 180° with an exposure time of 0.15 s per projection – leading to a total scan time per scan acquired of four minutes. A 500 μm cadmium tungstate (CdWO_4_) scintillator was used and the detector used was a PCO edge 5.5 CMOS detector. A fourfold optical magnification microscope system was used with a field of view of 4 × 3.5 mm and pixel size of 1.6 μm. With a propagation distance of 63.5 mm there was an intermediate amount of phase contrast. The attenuation data was reconstructed into 3D volumes of 2160 slices of 2560 × 2560 pixels each using a filtered back projection algorithm. These reconstructed 3D volumes had a 32-bit dynamic range upon reconstruction but were then down-sampled to 8-bit for ease of computational handling. During the 8-bit conversion, all images were down-sampled to the same fixed ranged which was set according to the minimum and maximum values found across all images. The list of SRXCT parameters is given in Table 1.

**Table 1:** Synchrotron data acquisition parameters for X-ray computed tomography (SRXCT) imaging and X-ray fluorescence (XRF) mapping

SRXCT		SRXRF	
Parameter	Value	Parameter	Value
DLS Beamline	I13–1	DLS Beamline	I18
Energy (keV)	15–20	Energy (keV)	5
Projections	1601	Edges excited	L2 and L3 iodine edges
Projection Exposure Time (s)	0.15	Pixel Size Resolution -Equivalent to Spot Size (μm)	10
Total scan time (min)	4	Monochromator	Si (111)
Scintillator	Cadmium tungstate (CdWO_4_)	Detector	Four-element Vortex Si drift detector
Detector	PCO edge 5.5 CMOS	X-ray flux	10^10^–10^11^ photons s^−1^
Magnification	Fourfold optical magnification	Standards used	Apatite mineral and spessartine garnet
Field of View (mm)	4 × 3.5	Calibrated Peaks	Mg, Al, Si, P, S, K, Ca, I, I (L alpha 2) and I (L beta 2).
Resulting Pixel Size Resolution (μm)	1.6		
Image Dimensions	2160 slices (2560 × 2560 pixels each)		

#### Soil and root SRXCT imaging

An SEM stub mount was used to mount the experimental systems for SRXCT imaging. This stub mount was modified to hold a syringe vertically tip-down by securing the tip with a luger connection. The design of this modified SEM stub is detailed in previous studies (Daly et al. 2016; Keyes et al. 2017a; Keyes et al. 2013). The basal tip of syringe which formed the bottom section of the root growth channel was inserted into this modified SEM stub and held tip-down such that the experimental system was vertical. The SRXCT imaging consisted of capturing 4 mm (diameter) by 3.5 mm (height) field of view regions of roots in soil at two different heights in the top section of the root growth channel. The top image was captured in a region between 13 and 16.5 mm below the base of top chamber (between 23.5 and 29 mm above the chamber containing contrast media) and the bottom image was captured in a region between 30 and 33.5 mm beneath the base of the top chamber (between 6.5 and 10 mm above the chamber containing contrast media) (Fig. 1d).

#### Leaf and stem SRXCT imaging

Following the imaging of roots in soil, sections of leaf and stem were excised from the maize plants and mounted for SRXCT imaging. Stem and leaf material was cut 5 mm above the soil and labelled according to a system where the central stem is labelled ‘Stem’, the most basal leaf is ‘Leaf 1’ and the second most basal leaf is ‘Leaf 2’ and so on. For the mount, a 2.5 mL syringe was cut in half along its length leaving the syringe tip intact. 3D printed blocks were then slotted into either end of the halved syringe (Fig. 1d). These 3D printed blocks had a flat surface facing out from the cutaway syringe. Wax was melted using a low heat soldering iron onto the flat surfaces of the blocks at either end of the syringe. To mount sections of leaf and stem this wax was reheated, the ends of the leaf material were held in the molten wax on the two blocks and as the wax cooled the leaves would become fixed in place ready for imaging (Fig. 1d). The syringe tip could then be placed into the modified SEM stub and thus be held vertical as described for the soil imaging system above. The imaged sections consisted of two different 3.5 mm lengths of leaf or stem material with a field of view diameter of 4 mm.

### SRXCT image analysis

The image analysis consisted of two main phases: manual segmentation of the root systems using Avizo 9.3.0 (Thermo Fisher Scientific, Waltham, MA, USA) and then all further analysis and quantification was completed using the FIJI distribution of ImageJ (Rueden et al. 2017; Schindelin et al. 2012).

The initial stage of image processing was segmenting the roots in the original absorption image stack. This segmentation was completed using the ‘manual segmentation’ tool. Each root present within an image stack was listed as a different ‘material’ and as such attributed a different colour label in the segmentation label mask. This allowed for individual roots to be analysed separately using a colour threshold to select masks for individual roots – for example, the primary root could be analysed separately. The segmented root stack was saved as a 3D .tif file.

#### Aerenchyma segmentation and area quantification

**Fig. 2:**
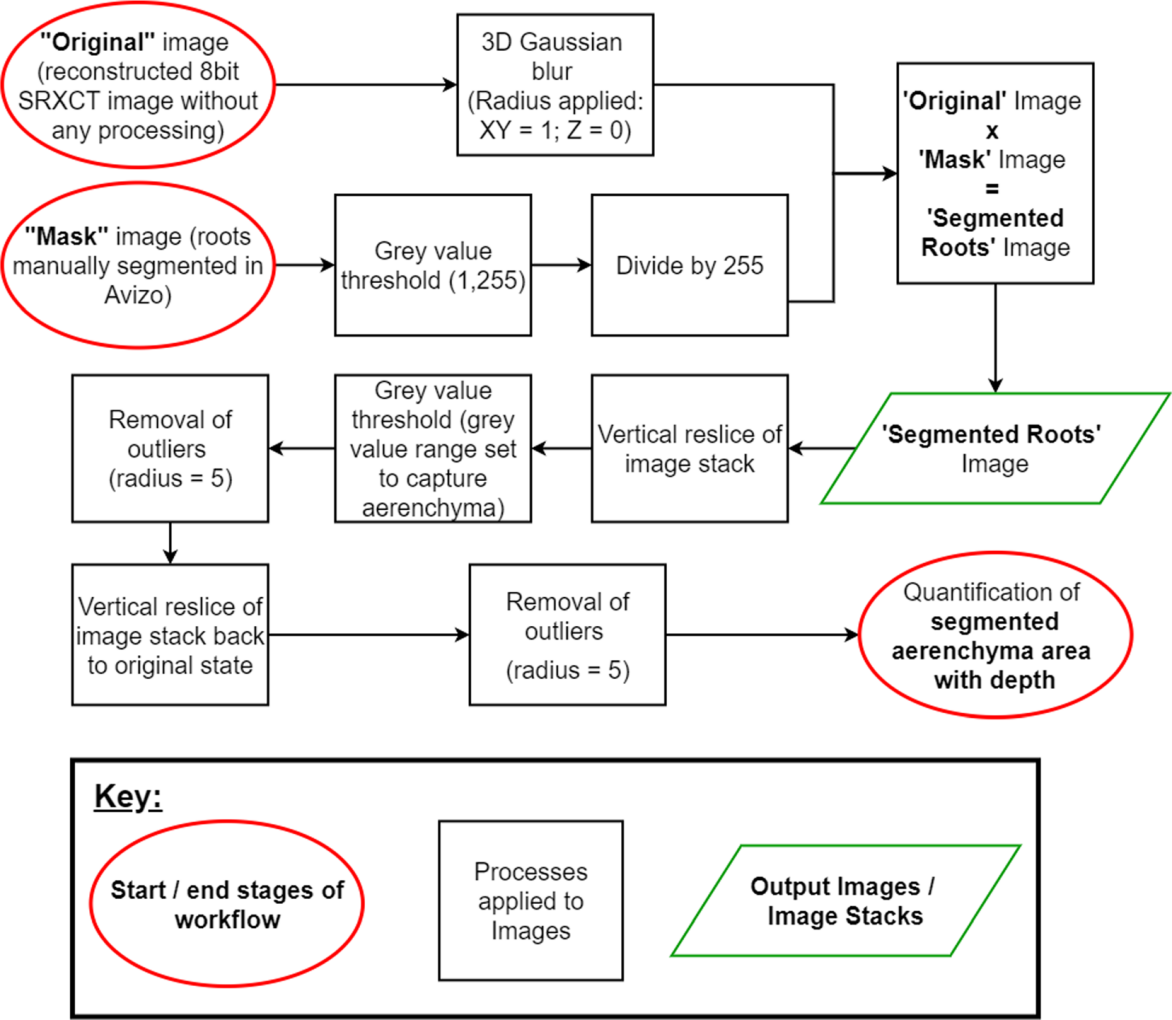
A flow chart describing the image processing algorithm used for segmenting the root aerenchyma. All image processing was completed in the Fiji distribution of ImageJ (Rueden et al. 2017; Schindelin et al. 2012) unless otherwise stated. A visual version of this flow chart is given in the supplementary information using visual examples of each step (Supplementary Fig. S2)

Next, the aerenchyma was segmented and the cross-sectional area of aerenchyma was quantified. This was particularly focused on the primary roots as many of the smaller secondary roots were either partially outside the region of interest (ROI) of the image stacks or at the artefact-rich edges. This image processing was implemented using the FIJI distribution of ImageJ (Rueden et al. 2017; Schindelin et al. 2012) and the algorithm used is outlined in Fig. 2 (an alternative visual version using example images is also presented in the supplementary information - Supplementary Fig. S2). First, a threshold of 1–255 was applied to the label mask image of the roots. This removed the background pixels with a grey value of zero. This image was then converted to 8bit from 8bit colour and the resulting image stack was divided by 255 to provide a mask for the segmented roots. The ‘Gaussian Blur (3D)’ tool was applied to the original unsegmented image stack using a radius of 1 in X and Y horizontal dimensions and 0 in the vertical Z dimension. The mask stack for the segmented roots was then multiplied by the original image stack to produce an image stack containing just the segmented roots.

**Fig. 3:**
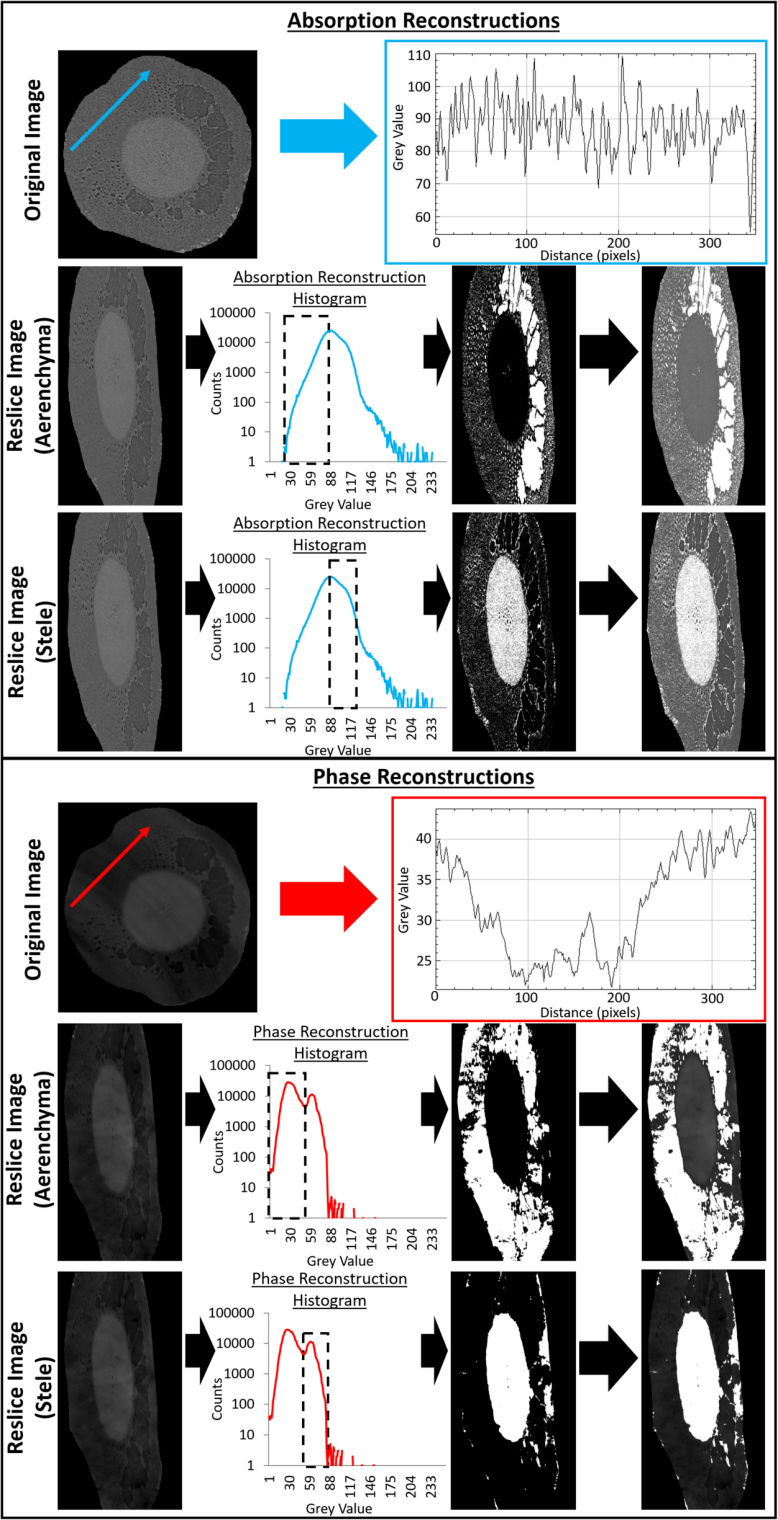
A comparison between the merits of absorption and phase reconstruction for segmentation of root anatomical features. Absorption images enable more reliable thresholding of aerenchyma whereas phase images enable more reliable thresholding of the stele. Ring artefacts complicated segmentation in the phase images to a greater extent than in absorption images

Next, the new image stack, containing just the segmented roots, was horizontally resliced using the ‘Reslice’ tool. This reduced the effects of ring artefacts on aerenchyma segmentation. The effect of these ring artefacts on grey values in the cortex can be seen in Fig. 3. A grey value threshold was applied to the resliced stack to segment voxels containing the aerenchyma. The grey value range used for this threshold was set from 1 to an upper limit of 10 beneath the modal grey value (the same as displayed in Fig. 3). The ‘Remove Outliers’ tool was applied to the image stack with a radius of 5 to remove some over-segmented noise. The ‘Reslice’ tool was then used to return the stack to the original orientation. Following this, the ‘Remove Outliers’ tool was applied again with a radius of 5 pixels to remove remaining over-segmented noise from the stack. The aerenchyma cross-sectional area was then quantified in each slice using the ‘Measure’ tool.

#### Quantifying stele Grey value with depth

Average grey value in cross sectional areas of the root stele was quantified with depth in the main roots as an indicator of contrast media presence. To achieve this, first the stele of the main roots in each original unsegmented image stack were manually segmented as a mask using the ‘manual segmentation’ tool in Avizo 9.3.0. The segmented root ‘Stele Mask’ image stack was saved as a 32bit 3D .tif file.

The next stage was quantification of the average grey value which was implemented using the FIJI distribution of ImageJ (Rueden et al. 2017; Schindelin et al. 2012). As with the aerenchyma segmentation, a new image stack which contained only the voxels of the stele of main roots was generated by multiplying the original unsegmented image stack with the Stele Mask image. The background voxels in the new image stack were assigned a grey value of 0. Finally, the mean grey value within the range 1 to 255 was calculated for each slice sequentially using the ‘Measure’ tool. This range was imposed on the mean grey value measurement to remove background pixels.

#### Quantifying cortex Grey value with depth

As with the other analyses, the quantification of the grey value of the cortex was implemented using the FIJI distribution of ImageJ (Rueden et al. 2017; Schindelin et al. 2012). First, the binarised root stele mask was subtracted from the segmented root stack (containing only the root and not the soil) using the ‘Image Calculator’ tool. This produced a new image stack which only contained voxels of the main root cortex. As with the stele grey value quantification, the background voxels in the image stack were assigned a grey value of 0. Finally, the mean grey value within the range 1 to 255 was calculated for each slice sequentially using the ‘Measure’ tool.

### Synchrotron X-ray fluorescence mapping and analysis

**Fig. 4:**
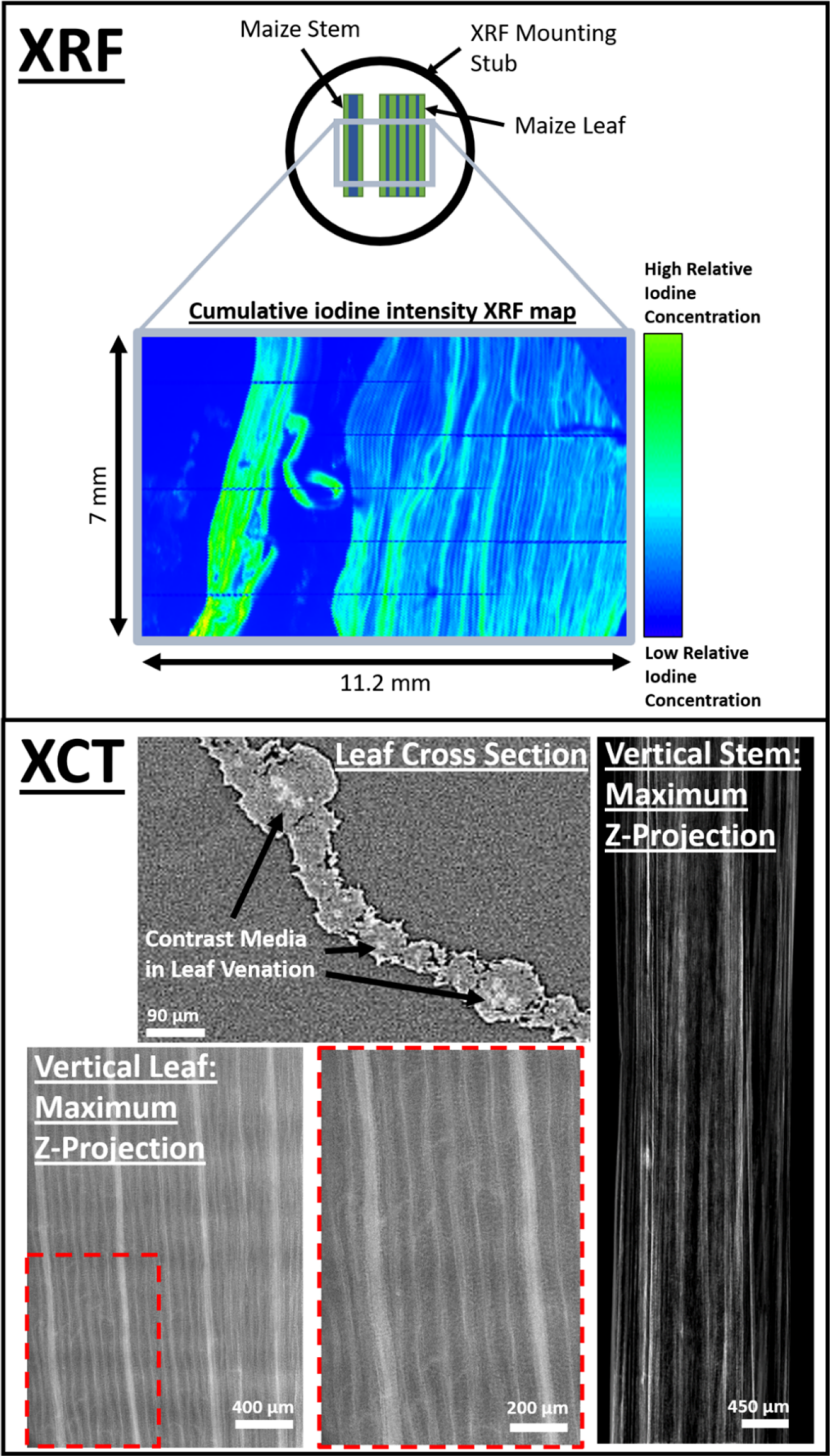
At the top is the SRXRF map of cumulative iodine intensity in stem and leaf material from ‘Maize 2’. The SRXRF panel displays a schematic diagram of the stem and leaf material mounted onto an ‘SEM stub’ for mapping indicating the region that was mapped (top of panel) and also the relative cumulative iodine SRXRF intensity map of the stem and leaf material (bottom of panel). On the bottom are SRXCT images displaying that contrast media was present in leaf venation and visible as the pixels of higher grey values. The SRXCT panel displays a leaf cross sectional slice (top) and a vertical reslice of a leaf which has undergone a maximum z-projection (bottom). In the vertical reslice images of the leaf is is possible to observe individual leaf vessels

The primary purpose of the XRF analysis was to observe where iodinated contrast media had accumulated in the plant in a cumulative iodine map produced from the XRF dataset which would allow visual observation (Fig. 4). Once SRXCT imaging had concluded, the plant material was flash frozen using a liquid nitrogen bath and then kept in a − 80 °C freezer. Five days after the conclusion of the SRXCT imaging a length of Stem and Leaf 1 of Maize 2 were removed from the freezer and were mounted side by side using a sticky carbon SEM stub (Fig. 4). The XRF map was captured at I18 at the Diamond Light Source synchrotron, Didcot, UK. To capture the XRF map the SEM stub was mounted on an x-y-z stage, rastered relative to the fixed incident beam. In order to obtain signal of low Z elements, the samples were held under a helium atmosphere. This beamline at DLS uses Kirkpatrick-Baez mirrors to produce spot sizes of 2–10 μm and the pixel size was chosen to be 10 μm. A Si(111) monochromator was used to select the incident beam energy and a four-element Vortex Si drift detector was used to detect X-rays. X-ray flux was estimated to be 10^10^–10^11^ photons s^−1^, the energy used was 5 keV and both the L2 and L3 edges of iodine were excited. The full energy dispersive spectrum was recorded for each pixel of the XRF map and the completed map was saved as an .nxs file. Fitting was undertaken using the freeware PyMCA X-ray Fluorescence Toolkit (Sole et al. 2007) from fundamental parameters of the experiment using standards of apatite mineral and spessartine garnet with known elemental concentrations for calibration. A table of SRXRF parameters is given in Table 1.

The analysis of the XRF maps was implemented using the PyMCA X-ray Fluorescence Toolkit (Sole et al. 2007). Reference peaks were calibrated for the following elements: Mg, Al, Si, P, S, K, Ca, I, I (L alpha 2) and I (L beta 2). The cumulative iodine map was then produced from the dataset to allow visual observations (Fig. 4).

## Results

### Confirmation of iodine induced contrast

**Fig. 5:**
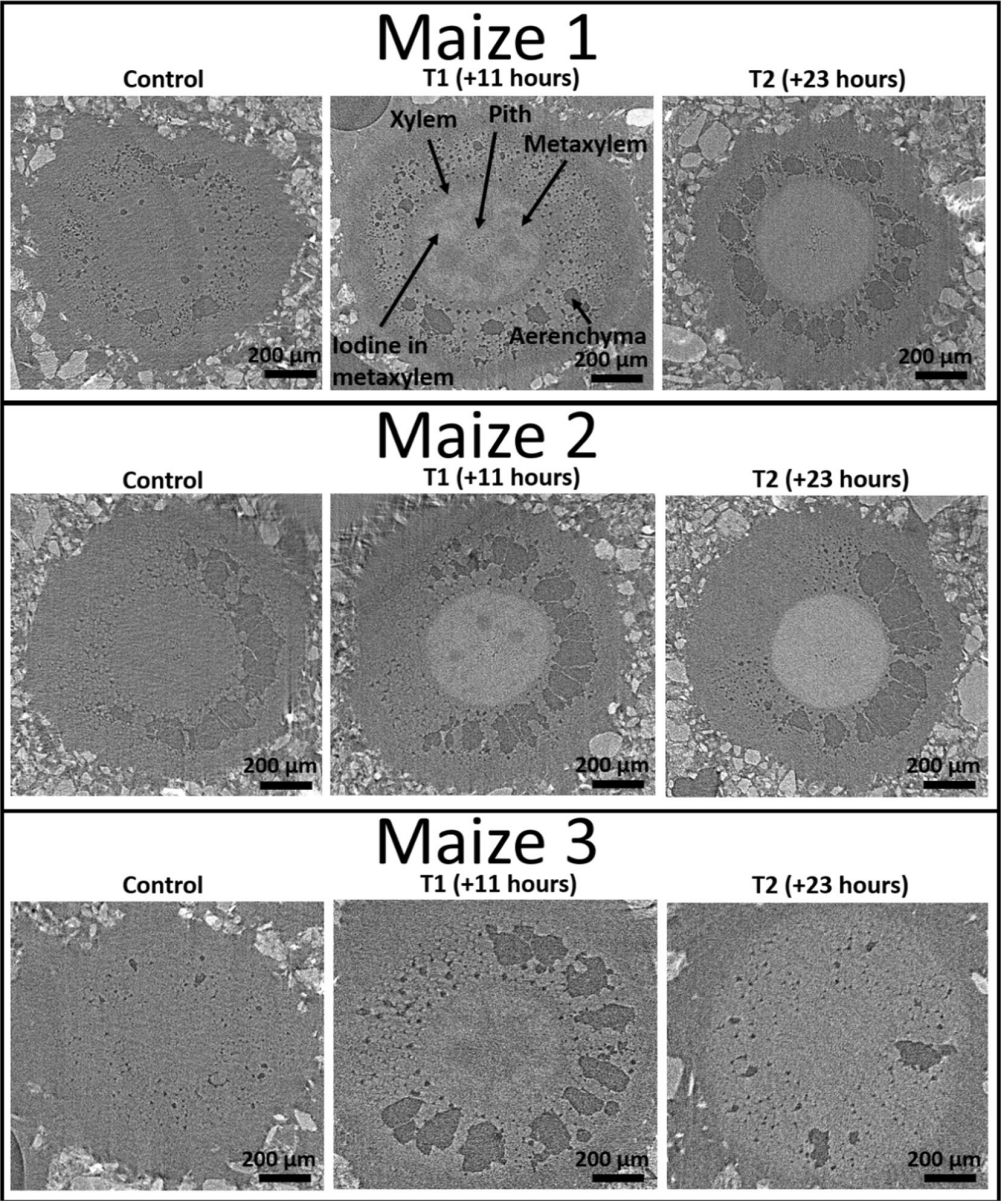
A collection of comparisons of SRXCT images of the main maize roots before the addition of the contrast media (Control), 11 h after the addition of contrast media (T1) and 23 h after the addition of contrast media (T2). Comparisons are made for each of the three replicates: Maize 1, Maize 2 and Maize 3. Contrast media is visible as the pixels of higher grey values and background pixels are lower in grey value. The contrast media enabled visualisation of anatomical root features otherwise not easily observable in SRXCT images such as xylem and metaxylem. In particular, the additional contrast in the plant vasculature provided by the contrast media means that metaxylem both containing contrast media and which does not contain contrast media can be visualised. With the addition of contrast media the stele, in the centre of the root where the root vasculature can be found, considerably contrasts against the cortex material outside whereas without contrast media the stele is not easily distinguished. Fibres can also be visualised in the aerenchyma

The contrast media is visible in comparisons of SRXCT images of the main maize roots before the addition of the contrast media (Control) and 11 h after the addition of contrast media (T1) as regions of comparatively higher grey values (Fig. 5). The stele, in the centre of the root where the root vasculature can be found, can be observed with the use of contrast media. Considerable contrast of the stele against the cortex material can be seen with the contrast media (Figs. 7 and 8**and** Supplementary Figs. S7 and S8), whereas without contrast media the stele is not distinguishable from the cortex (Fig. 5). In addition, the contrast media enabled visualisation of finer scale anatomical root features such as the xylem and metaxylem (Fig. 5). In particular, the additional contrast in the plant vasculature provided by the contrast media means that it is possible for metaxylem to be visualised when it both contains contrast media and when it does not contain contrast media – a feature which is otherwise difficult to observe using SRXCT owing to the limited contrast to noise ratio within root material. Whilst the stele can be observed in both absorption and phase reconstructions, the stele is more accurately segmented using thresholding in phase images (Fig. 3). In phase images the stele is represented by a clear second peak in grey value histograms whereas, in absorption images the peak in grey value histograms which represents the stele is only visible as a non-discrete ‘shoulder’ (Fig. 3). In contrast, the aerenchyma is more accurately segmented in absorption rather than phase reconstructions (Fig. 3). The grey value ranges of the histograms presented in Fig. 3 with zero counts would have included the soil in non-segmented image stacks.

The XRF map which was produced for the mounted section of leaf and stem material from Maize 2 confirmed that iodine had travelled through the plant vasculature from the roots to the leaf and stem material. This is indicated by the high cumulative iodine concentration which could be observed in both the stem and leaf material in the iodine map of the sample (Fig. 4). The iodine appeared to be particularly concentrated in the vasculature of the leaves and stem, i.e., in the parallel leaf venation. This high concentration was visualised as the green regions in Fig. 4. This accumulation of contrast media in the leaf venation is also visible in the leaf SRXCT data in both cross sectional images and in vertical reslices of image stacks which have undergone a maximum z-projection (Fig. 4).

### Average stele Grey value

**Fig. 6:**
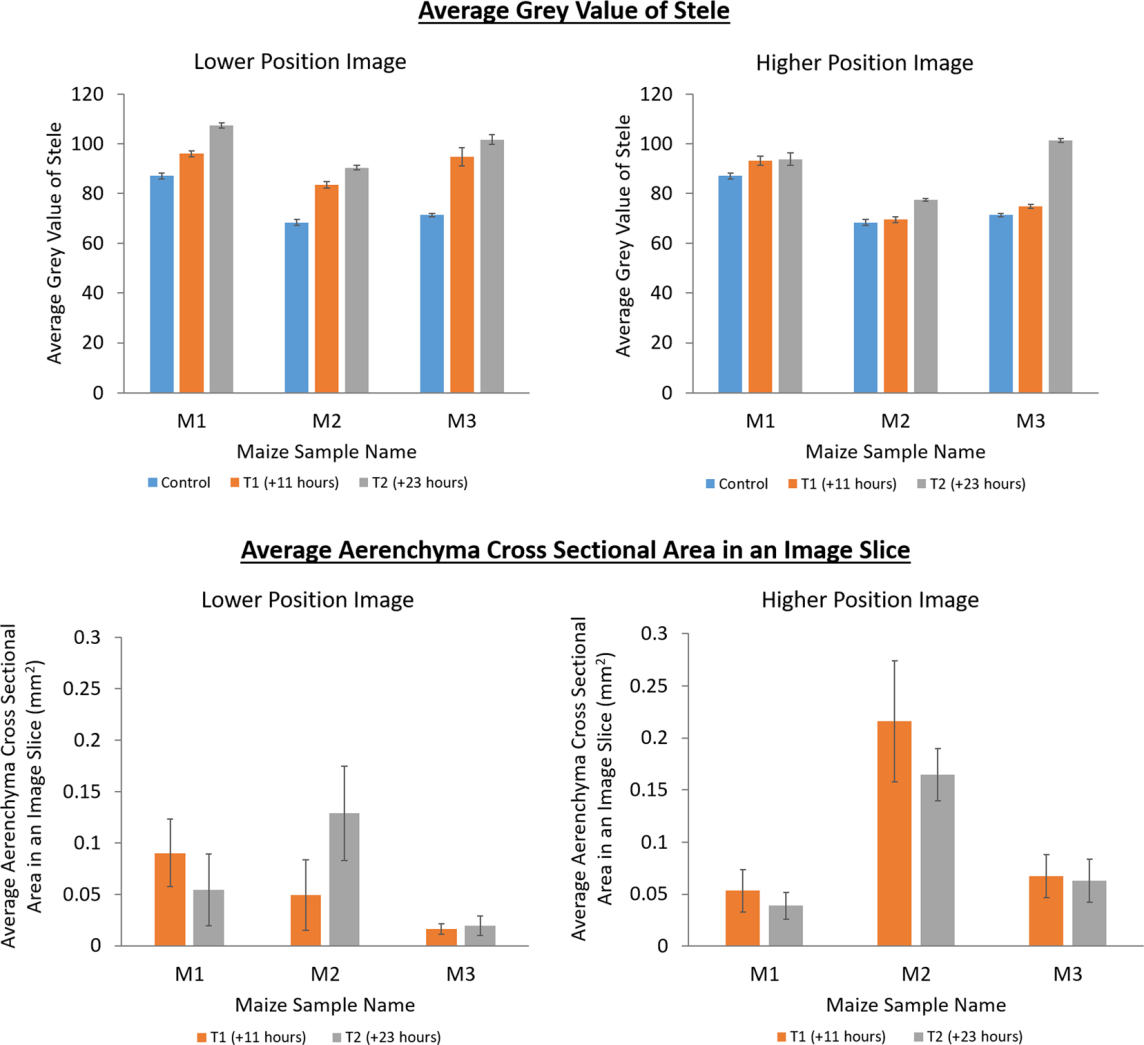
The average grey value along lengths of root stele in main roots (top) and the average aerenchyma cross sectional area (bottom) at different imaging time points. This is presented for both the lower image positions (left) and the higher image position (right). The control time point (blue) was prior to the addition of contrast media, the T1 time point (orange) was 11 h after the addition of contrast media and the T2 time point (grey) was 23 h after the addition of the contrast media. Control time points are included for the average stele grey value plots as a baseline for comparison and to demonstrate that the contrast media increased the grey value in the stele relative to the control. This is not relevant to the aerenchyma cross sectional area plots as these are simply to demonstrate that there is no significant differences in cross sectional area between T1 and T2 - these are the relevant time points for the comparisons made in Fig. 7 and Fig. 8. Standard error bars are also provided

The average grey value along the length of the primary root stele with time increased (Figs. 6). This appeared to be true of the higher and lower imaged sections of the primary roots in all three replicates. In the lower imaged section the average grey values increased from 96, 83.4 and 94.8 at T1 to 107.3, 90.43 and 101.6 at T2 for M1, M2 and M3, respectively (Figs. 6). In the higher imaged section the average grey values increased from 93.3, 69.5 and 74.8 at T1 to 93.8, 77.5 and 101.3 at T2 for M1, M2 and M3, respectively (Figs. 6). For all three replicates the initial average grey value in the main root stele was similar between the lower and higher imaged sections at the control time point, i.e., before contrast media was added. However, at T1 and T2 (11 and 23 h after the addition of contrast media, respectively) the average grey value increased though by a smaller amount in the higher imaged section of the primary roots than the lower imaged section (Fig. 6).

**Fig. 7:**
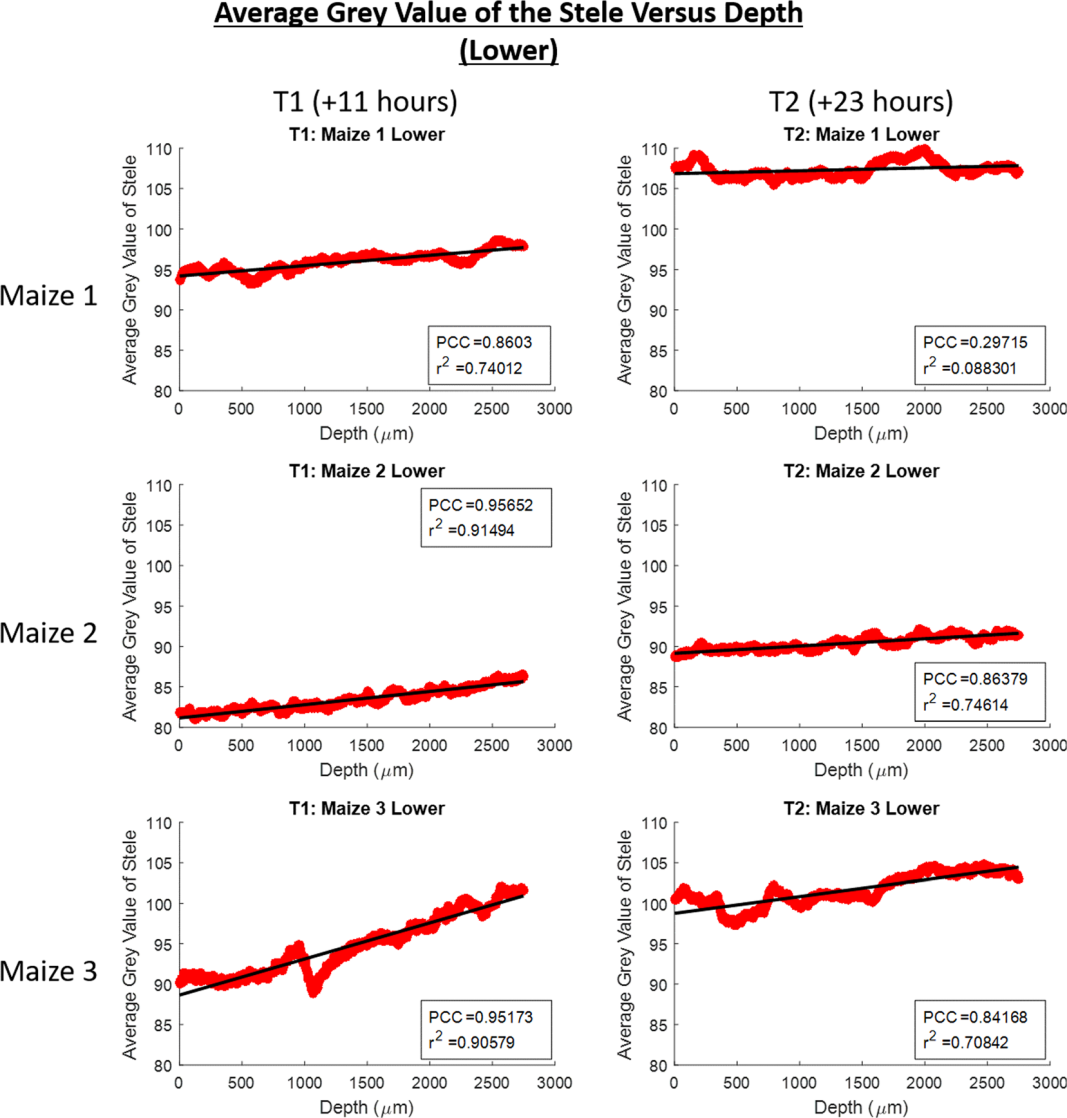
Scatter plots of average grey value of the root stele versus depth down the root growth channel for the lower imaged section of roots. Zero on the depth axis is the top of the image. T1 is the time point for scans acquired 11 h after the addition of contrast media and T2 is the time point for scans acquired 23 h after the addition of contrast media. Maize 1, Maize 2 and Maize 3 are the names of each of the plant samples. The Pearson correlation coefficient, r^2^ value and linear best fit lines are provided for each scatter plot

**Fig. 8:**
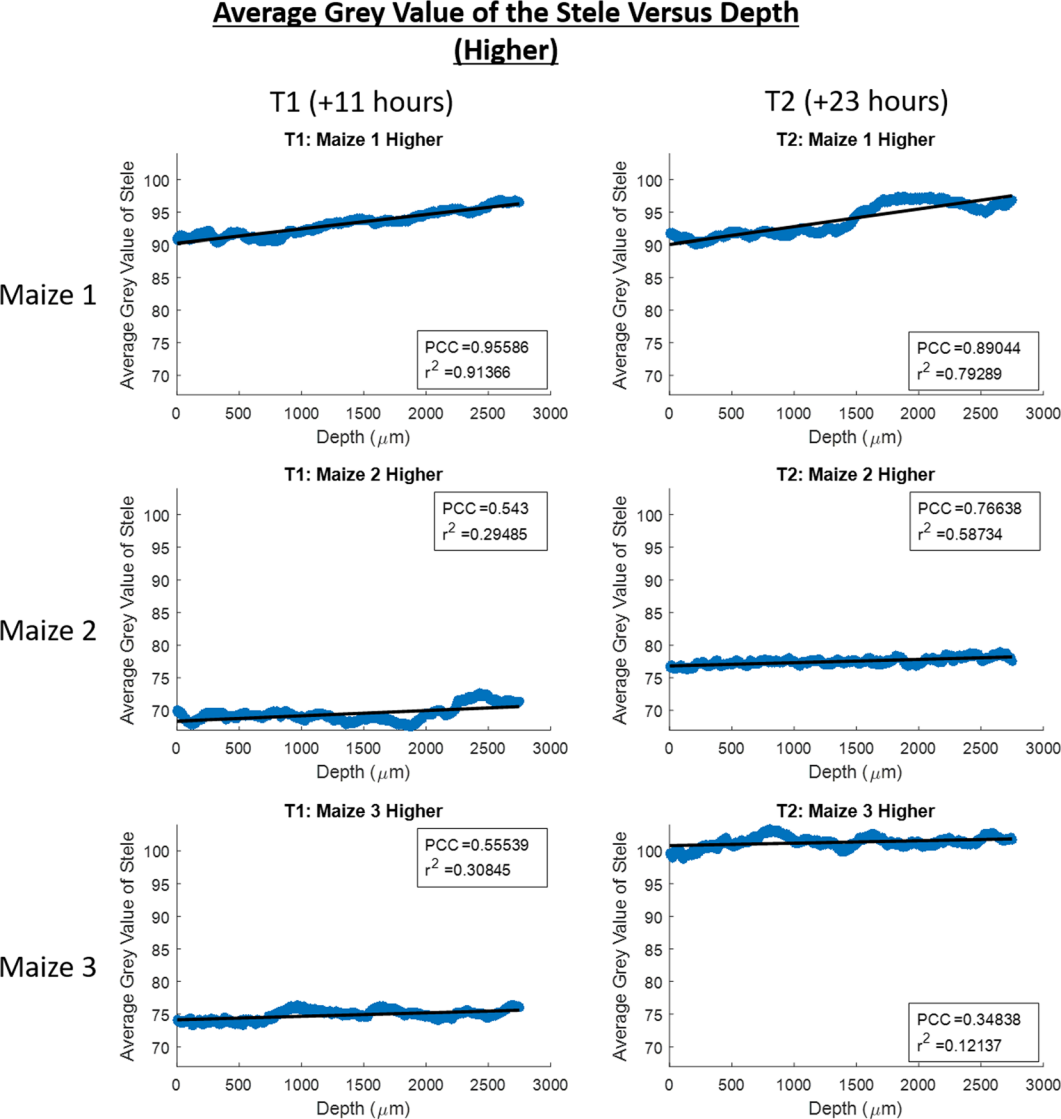
Scatter plots of average grey value of the root stele versus depth down the root growth channel for the higher imaged section of roots. Zero on the depth axis is the top of the image. T1 is the time point for scans acquired 11 h after the addition of contrast media and T2 is the time point for scans acquired 23 h after the addition of contrast media. Maize 1, Maize 2 and Maize 3 are the names of each of the plant samples. The Pearson correlation coefficient, r^2^ value and linear best fit lines are provided for each scatter plot

This was explored in greater detail where the grey value in the stele with depth was investigated within image stacks (Figs. 7 and 8). In all image stacks the grey value was higher in the stele at greater depths (nearer to the basal tip of the roots – Figs. 7 and 8). Additionally, the grey value in the lower sections of root increased at a greater rate with depth (Fig. 7) than in the higher sections (Fig. 8). The changes in the average stele grey value with depth are substantial in comparison with the smaller changes in cortex grey value with depth where there appears to be no discernible trend present across the data (Supplementary Figs. S7 and S8).

### Correlating stele grey value and aerenchyma cross sectional area over depth

In ten of the twelve comparisons a Pearson correlation coefficient (PCC) analysis reported a negative correlation between the average grey value of primary root stele with aerenchyma cross sectional area over all root depths (Table 2**,** Supplementary Figs. S3 and S4). Of those ten which featured a negative correlation, six could be considered to report a comparatively strong negative correlation (PCC < −0.6). There was no clear relationship between the strength and direction (positive or negative) of the reported PCC and whether the comparison is made in the lower or higher of the imaged root sections (Table 2). There was also no clear relationship between the strength and direction (positive or negative) of the reported PCC and whether the comparison is made at T1 or T2 (Table 2). This is reflected in the variation of aerenchyma cross sectional area with depth where no apparent consistent trends are present between imaging time points or image position (Fig. 6 and Supplementary Figs. S5 and S6).

**Table 2:** Pearson Correlation Coefficient of average grey value of root stele versus aerenchyma cross sectional are over depth. T1 is the time point for scans acquired 11 h after the addition of contrast media and T2 is the time point for scans acquired 23 h after the addition of contrast media. Position refers to whether the data is from the upper or lower imaged region of root length captured at different heights. Upper means the region of root imaged which was nearer to the above soil material and Lower means the region of root imaged which was nearer to the root tip. M1, M2 and M3 are the names of each of the plant samples. Full scatter plots of this data for the lower and higher image positions can be found in Supplementary Figs. S3 and S4 respectively

Time - Position	M1	M2	M3
T1 - Lower	−0.415	0.188	−0.301
T1 - Upper	−0.662	−0.349	−0.843
T2 - Lower	−0.712	−0.381	−0.769
T2 - Upper	0.396	−0.839	−0.773

The correlation plots indicated that for the two comparisons which reported a weak positive correlation, Maize1-T2-Higher and Maize3-T2-Lower, the full data was variable with a low r^2^ value of 0.15 or below (Supplementary Figs. S3 and S4). The correlation plots for the six comparisons which reported a strong negative correlation (PCC < −0.6) also indicated evidence of a strong negative correlation with all reporting an r^2^ value of 0.4 or above (Supplementary Figs. S3 and S4). It should be noted that for Maize3-Lower the correlation plots at both T1 and T2 displayed considerably smaller values for the aerenchyma cross sectional area - approximately an order of magnitude smaller than in other samples (Fig. 6**)**.

## Discussion

The aim of this study was to develop an experimental system which enabled iodinated contrast media to be supplied directly to plant roots for uptake and translocation compatible with time-resolved SRXCT to facilitate in vivo imaging of plant root material in soil. This was intended to overcome issues of low contrast to noise within XCT images of plant roots and soil environments which can complicate image processing and can result in the loss of physiological/ anatomical information (Mooney et al. 2012). The experimental setup performed its intended purpose enabling in vivo imaging of live root systems and capturing the translocation of the contrast media through root vasculature into the leaves, revealing additional root anatomical information.

The successful translocation of the contrast media from the roots in to the leaves, evidenced by both SRXCT and SRXRF data (Fig. 4), indicated that this system performed equally well to Keyes et al.*,* (2017b) with regard to achieving contrast media distribution in in vivo plant material. However, our system provided some additional benefits, i.e., enabling visualisation of anatomical features of the root system (Fig. 5). We were able to capture details such as the position of the stele, the vascular structure, fibres in aerenchyma and identify the accumulation of contrast media in leaf / stem venation (Figs. 4 and 5). In particular, examples of xylem and metaxylem can be observed both containing contrast media and also empty within the same image stack identifying the location of different vascular features (Fig. 5). This was possible in both the phase and absorption reconstructions, though each offered different benefits with regard to segmenting specific root features. In absorption images it was possible to more accurately to segment aerenchyma, as ring artefacts in the phase images make thresholding more complex (Fig. 3). However, the presence of a discrete second peak in the grey value histogram of phase images meant the stele could be segmented with greater accuracy using phase reconstructions (Fig. 3). Since contrast media accumulated in the vasculature the position of the leaf venation could be mapped using both SRXCT and SRXRF (Fig. 4) – this was not possible in SRXCT images without the application of contrast media. This also meant that we were able to capture individual leaf vessels in both the SRXCT and SRXRF images (Fig. 4). Such features can be captured in great detail and at high magnification using established microscopy techniques. However, these images are limited to 2D and often requires the tissue be removed from the plant and stained rather than remaining intact in soil (Hall and Hawes 2012) – as such, time resolved imaging of live tissue is not practicable. Whilst microscopy imaging may capture these features in greater detail than the SRXCT images of this study, SRXCT enables 3D time-resolved imaging of these features in soil which remain sufficiently clear so as to be segmented using simple grey value thresholds (Fig. 3). Additionally, with the development of nanoparticulate contrast media for use in soil (Scotson et al. 2019), it is possible that in future the in vivo anatomical imaging achievable with our experimental system could be further enhanced with the use of nanoparticles functionalised to accumulate on particular plant tissues or organs. Particularly with the use of nanoparticles of approximately 3.5 nm in size, which have previously been observed to pass into roots and then be translocated throughout the plant to the leaves (Sabo-Attwood et al. 2012).

A further benefit of our system is that it involves application of the contrast media directly to the root. This could be of importance given the potential for iodinated contrast media to have osmotoxic effects over time, as identified by Keyes et al., (2017b). By applying the contrast media directly to the root it is possible to capture the contrast media within root tissues in soil without the time delay necessary for translocation from the leaf to the roots as in the Keyes et al., (2017b) system (all the while osmotoxic effects may be developing and plant health may be deteriorating). Using our system it would also be possible to study further dynamics of root and soil interactions, such as the effect of soil porosity or soil compaction on soil-water uptake. This could be achieved by applying the contrast agent through a partially saturated soil rather than submerging the root in a contrast media solution. Such an approach of application via partially saturated soil could be paired with studies which seek to capture root-water interactions, such as Scotson et al., (2020), to monitor effects of root water uptake on soil pore water.

Our system was also capable of tracking the translocation of the contrast media through the root vasculature with time. This translocation was observed as the change in grey value over time in the lower and higher imaged sections of the roots (Fig. 6). Over time as more contrast media entered the root stele, the grey value inside the root in the lower imaged section increased and then more gradually the grey value also increased in the higher imaged section of the root as the contrast media was translocated further up the vasculature (Fig. 6). This translocation of the contrast media into the stele and up the root vasculature was further evidenced by the average grey value of the stele with depth in individual image stacks. Within all individual image stacks the average grey value of the stele increased with time and was greater with depth (Figs. 7 and 8). However, this increase was again more gradual within the image stacks captured at the higher position (Fig. 8) – potentially a result of the time required for the contrast media to translocate up through the root from the lower to higher imaging position. Whilst the grey value within the stele does increase with time in all instances, there is some variation within the rate increase - possibly a result of slight variations in root growth and development between plants. Given that we assume that the grey value in the stele is indicative of the plant solute uptake rate, more developed roots, or those plants with longer lengths of root submerged into the contrast media, would likely have a faster rate of contrast media uptake (Gardner 1991; Roose and Schnepf 2008).

In contrast, the average grey value within the cortex showed no such trend of consistently increasing as contrast media was translocated further up the root material (Supplementary Fig. S7 and S8) which would suggest that the upwards translocation of the contrast media did take place through the vasculature in the stele, rather than in an apoplastic manner through the cortex. Were the contrast media to be translocated upward through the cortex it would be expected that there would be a trend of average grey values in the cortex increasing with time, as is observed in the stele (Figs. 7 and 8), though this trend is not present (Supplementary Fig. S7 and S8). Where there are variations in the cortex grey value with depth it is possible that this is a result of contrast media leaking from the stele through damaged endodermis tissues – Keyes et al., (2017b) observed similar leaking of contrast media from the stele into the cortex which was thought to be a result of osmotic stress effects of the contrast media over time. This is likely to be further exacerbated with prolonged exposure, for example where time is required for contrast media to translocate from leaves to the root system as in Keyes et al., (2017b), and thus the benefit of our system delivering contrast media immediately to the roots is reinforced.

The grey value data could then be correlated with other anatomical characteristics of the roots such as the aerenchyma cross sectional area with depth (Supplementary Figs. S3 and S4). In half of the image stacks we were able to capture strong negative correlations between aerenchyma cross sectional area and grey value (Table 2 and Supplementary Figs. S3 and S4), despite little evidence of a consistent trend in aerenchyma cross sectional area with depth (Supplementary Figs. S5 and S6). This potentially indicates that aerenchyma may act as a barrier to solutes moving through the cortex and into the stele as has previously been observed (Fan et al. 2007; Hu et al. 2014). Owing to the exploratory nature of this study, the replicate number is too small to rigorously answer biological questions relating to the effect of aerenchyma cross sectional area on the transport of solutes through the cortex and into the stele. However, we can present this an example avenue for future study using our experimental system. For example, if this system was used in larger studies with more replicates, such data could be used to parametrise models for plant solute or water transport, such as those of Payvandi et al. (2014), under different environmental conditions.

Iodinated contrast media has been used previously to study larger soil and plant systems and one future avenue for exploration could be upscaling the experimental system developed in this study and using it to capture solute movement through roots in considerably larger soil systems. Previous studies have investigated fluid flow through soil cores of 10 cm in diameter or larger using radiographic contrast media (Heijs et al. 1996; Larsbo et al. 2014; Scotson et al. 2020) and, were such methodologies to be combined with the principles of the experimental system used in this study, fluid flow and nutrient movement through both the soil and through the root system could be studied simultaneously. Such data could then be used to parameterise mathematical models of solute movement through soil to roots and then transport within the plants. Additionally, using larger soil systems would facilitate the use of larger and more mature plants which would likely tolerate contrast media better than juvenile plants. By applying the principles of our experimental system, contrast media could still be delivered immediately to the roots of larger plants without the increased time delay required by the system established by Keyes et al.*,* (2017b) for translocation of the contrast media from the leaves to the roots of larger plants. Not only could this enable fluid flow tracing through the root system, but it would also likely simplify segmentation of roots by increasing the contrast to noise ratio of the roots within larger soil samples which can otherwise present segmentation issues (Attix 2008; Mooney et al. 2012).

In addition to the opportunities outlined above, it is important to note that there are potential considerations in the use of this experimental system which could affect future applications and implementation. As mentioned, iodinated contrast media can induce osmotic stress effects over time resulting in internal plant tissue damage which may affect normal plant function and growth (Keyes et al. 2017b; Zhang et al. 2016). We would not have captured changes in root growth caused by the addition of contrast media as the contrast media was only applied to the roots for the imaging period of 24 h and the root tips themselves were not captured. The purpose of this investigation was to assess whether our system would facilitate contrast media uptake however, were growing root tips to be imaged, such as in the work of Keyes et al., (2017a), it would be possible to capture effects of contrast media on root growth over time. In order to mitigate these effects, non-ionic iodinated contrast media should be used, such as the iopamidol used in this investigation, as ionic alternatives have previously been observed to induce more significant osmotic stress effects in biological tissues (Haller and Hizoh 2004; Hasebroock and Serkova 2009; Heinrich et al. 2005; Sendeski 2011). Additionally, the osmolality, closely associated with osmotic shock effects (Lusic and Grinstaff 2013), of non-ionic contrast media has been reported to be at least four times lower than that of ionic contrast agents of comparable iodine concentrations (Aspelin 2006). Osmotoxic effects of iodinated contrast media could be further mitigated if using more dilute contrast media since more dilute contrast media is likely to draw less water from nearby plant cells (Lusic and Grinstaff 2013). It would therefore be worthwhile to conduct further optimisation studies to assess the necessary contrast media concentrations for achieving sufficient contrast within plant tissues whilst considering a minimisation of osmotoxic effects. However, it should be noted that, even when using diluted non-ionic contrast media, prolonged exposure to iodinated contrast media will still have implications for plant health - particularly where juvenile plants are used. Therefore, there are limitations on the duration of experiments which require continuous exposure of plants to contrast media. This perhaps reinforces that upscaling the experimental system to a size similar to Heijs et al., (1996), Larsbo et al., (2014) and Scotson et al., (2020) to support more mature plants, which are more stress tolerant (Choudhury and Kumar 1980; Derera et al. 1969; Singh et al. 2012), would be a positive progression of our system which could also aid mitigation of osmotoxic water stress effects of the contrast media.

In conclusion, we have demonstrated that our experimental system, which utilises contrast media applied to intact in vivo plant roots within soil, can enhance SRXCT imaging of the root system and elucidate details of the internal root structure. We confirmed the translocation of the contrast media throughout the plants with the novel use of both SRXCT and SRXRF imaging. With the application of the contrast media it was possible to visualise anatomical information including the stele, the root vasculature, fibres in the aerenchyma and the leaf venation. Examples were then provided of the features which could be studied using our system such as the effect of aerenchyma cross sectional area on fluid and nutrient transport in plant root systems. Our system could be used in future to study many different aspects of solute transport, root anatomy and structure, much of which would otherwise require microscopy of stained ex vivo plant tissues. The data from such studies could then be utilised to parameterise mathematical models of solute transport through varying internal root structures to investigate optimal root structure for efficient solute transport.

## Supplementary information

ESM 1 (DOCX 2412 kb)
