# Supplementary material for: Developing a system for in vivo imaging of maize roots containing iodinated contrast media in soil using synchrotron XCT and XRF
Source: Plant Soil. 2020 Dec 10;460(1-2):647–65. doi: 10.1007/s11104-020-04784-x (PMC8550435; doi:10.1007/s11104-020-04784-x)
Supplement: Supplementary file 1 — (DOCX 2412 kb) [file 11104_2020_4784_MOESM1_ESM.docx]

# Supplementary Information: Developing a System for In-Vivo Imaging of Maize Roots Containing Iodinated Contrast Media in Soil using Synchrotron XCT and XRF

## Supplementary Figures


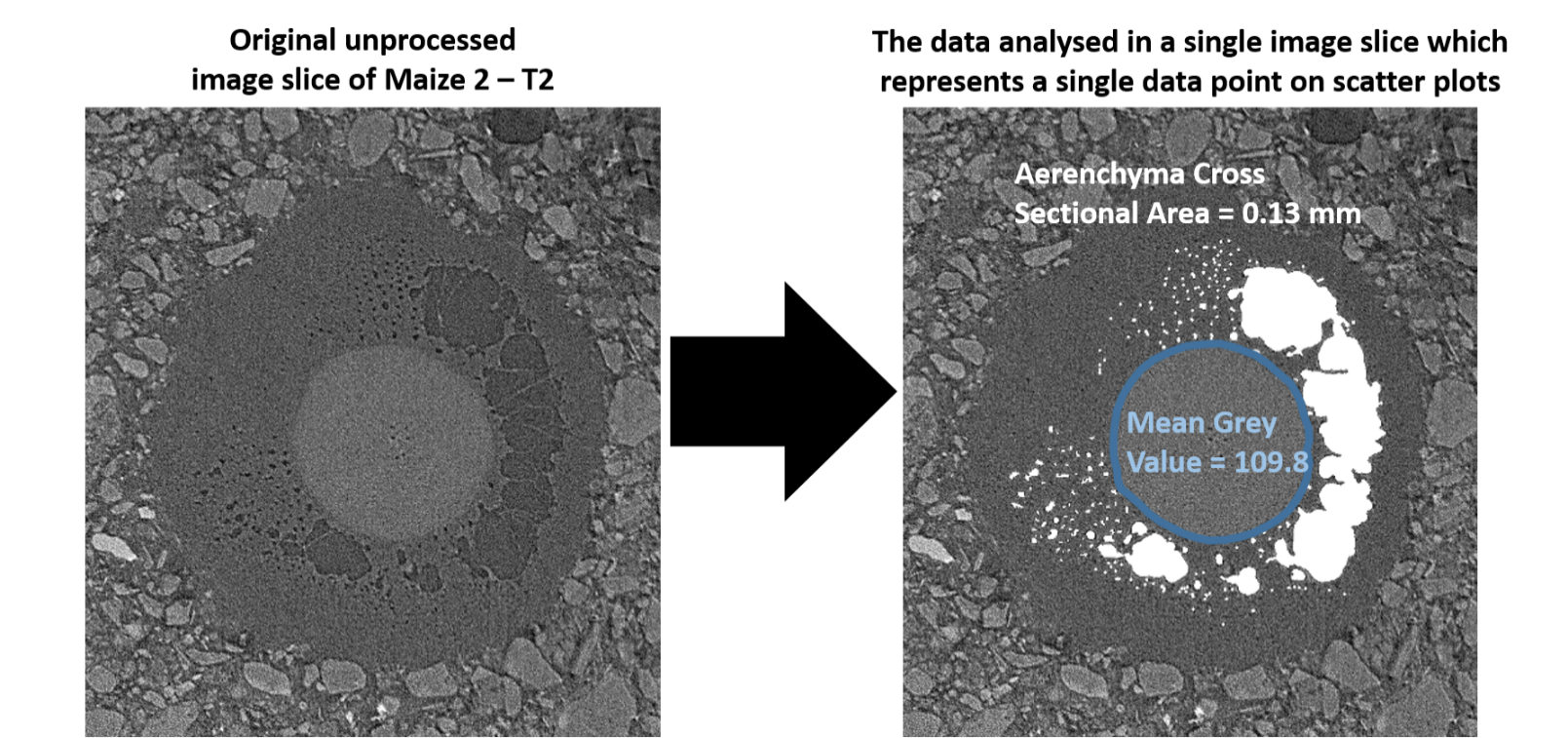


**Supplementary Figure S1.** An example of the data lifted from an image slice which is then represented by data points on the scatter plots of stele grey value and aerenchyma cross sectional area. A comparison is provided between an original unprocessed image (left) and an image displaying an overlay of the regions analysed for stele grey value and aerenchyma cross sectional area (right).


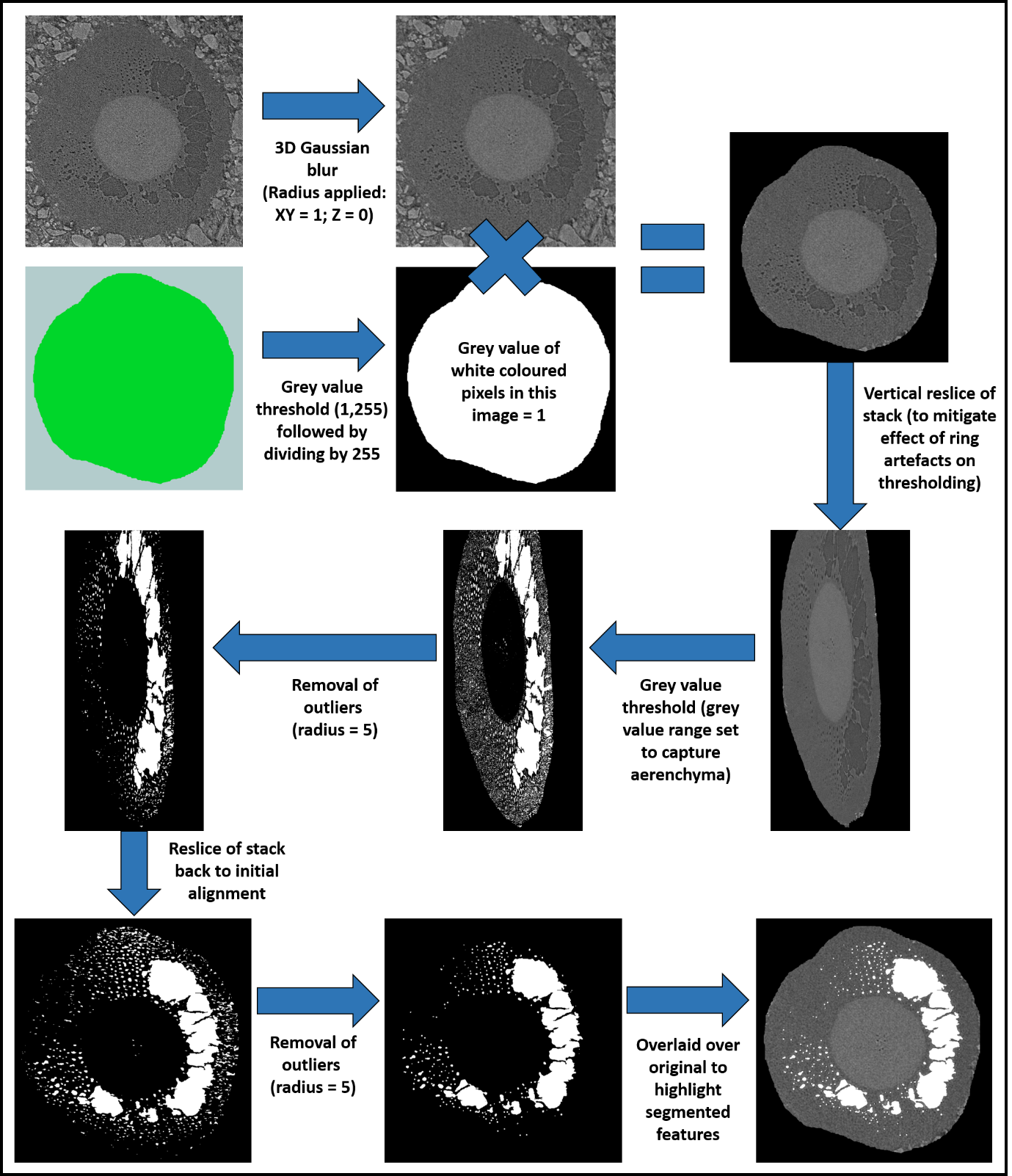


**Supplementary Figure S2.** A visual flow chart describing the image processing algorithm used for segmenting the root aerenchyma. The purpose of the vertical reslice step was to mitigate for the effects of ring artefacts on grey value thresholding. All image processing was completed in the Fiji distribution of ImageJ (Schindelin *et al.*, 2012; Rueden *et al.*, 2017) unless otherwise stated. A diagrammatic version of this flow chart is given in the main manuscript (**Figure 2**).

**
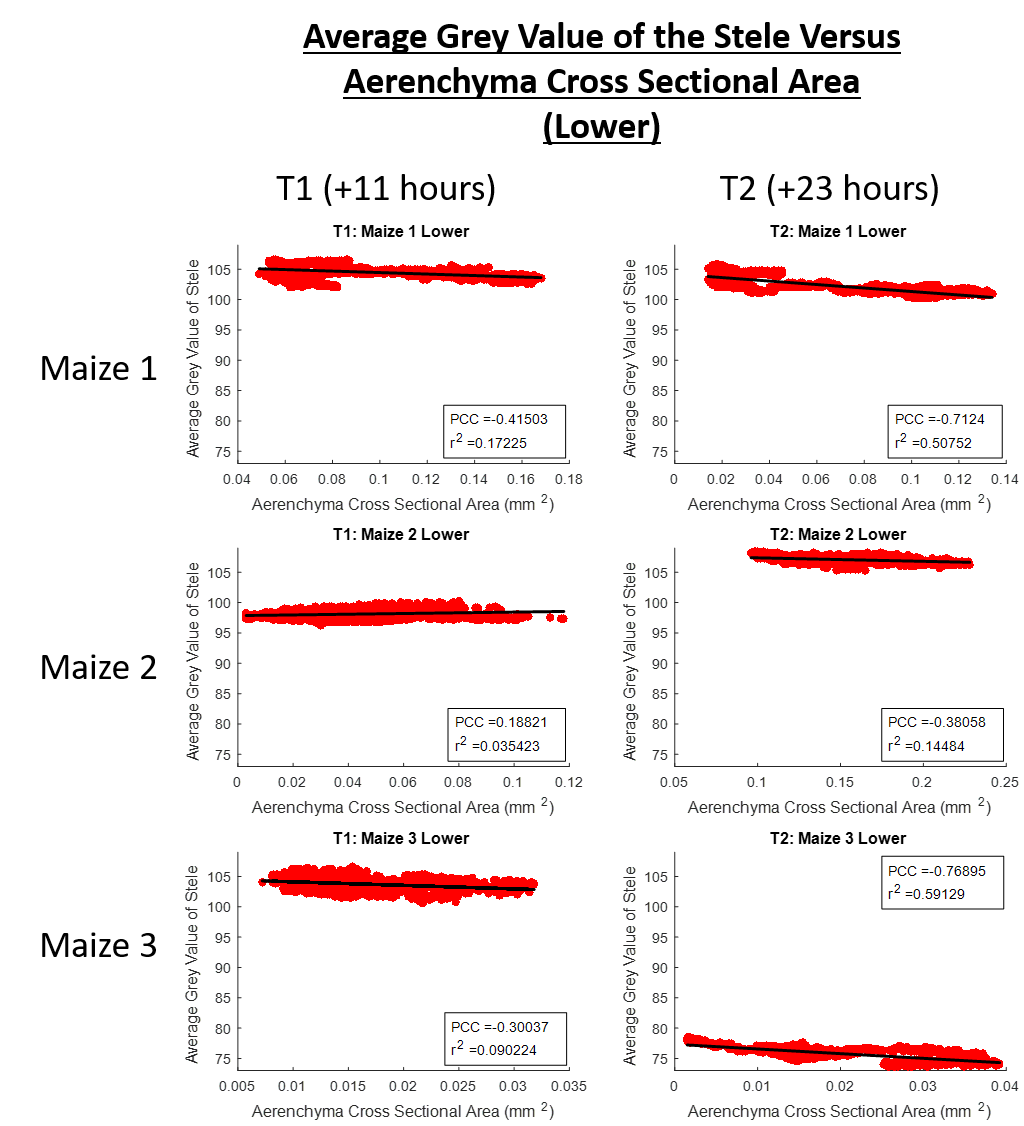
**

**Supplementary Figure S3.** Scatter plots of average grey value of root stele versus aerenchyma cross sectional area over depth for the lower imaged section of roots. Each individual data point on the scatter plots represents the average grey value of the stele and the aerenchyma cross sectional area recorded in a single slice from within the image stack (**Supplementary Figure S1**). T1 is the time point for scans acquired 11 hours after the addition of contrast media and T2 is the time point for scans acquired 23 hours after the addition of contrast media. Maize 1, Maize 2 and Maize 3 are the names of each of the plant samples. The Pearson correlation coefficient, r^2^ value and linear best fit lines are provided for each scatter plot.

**
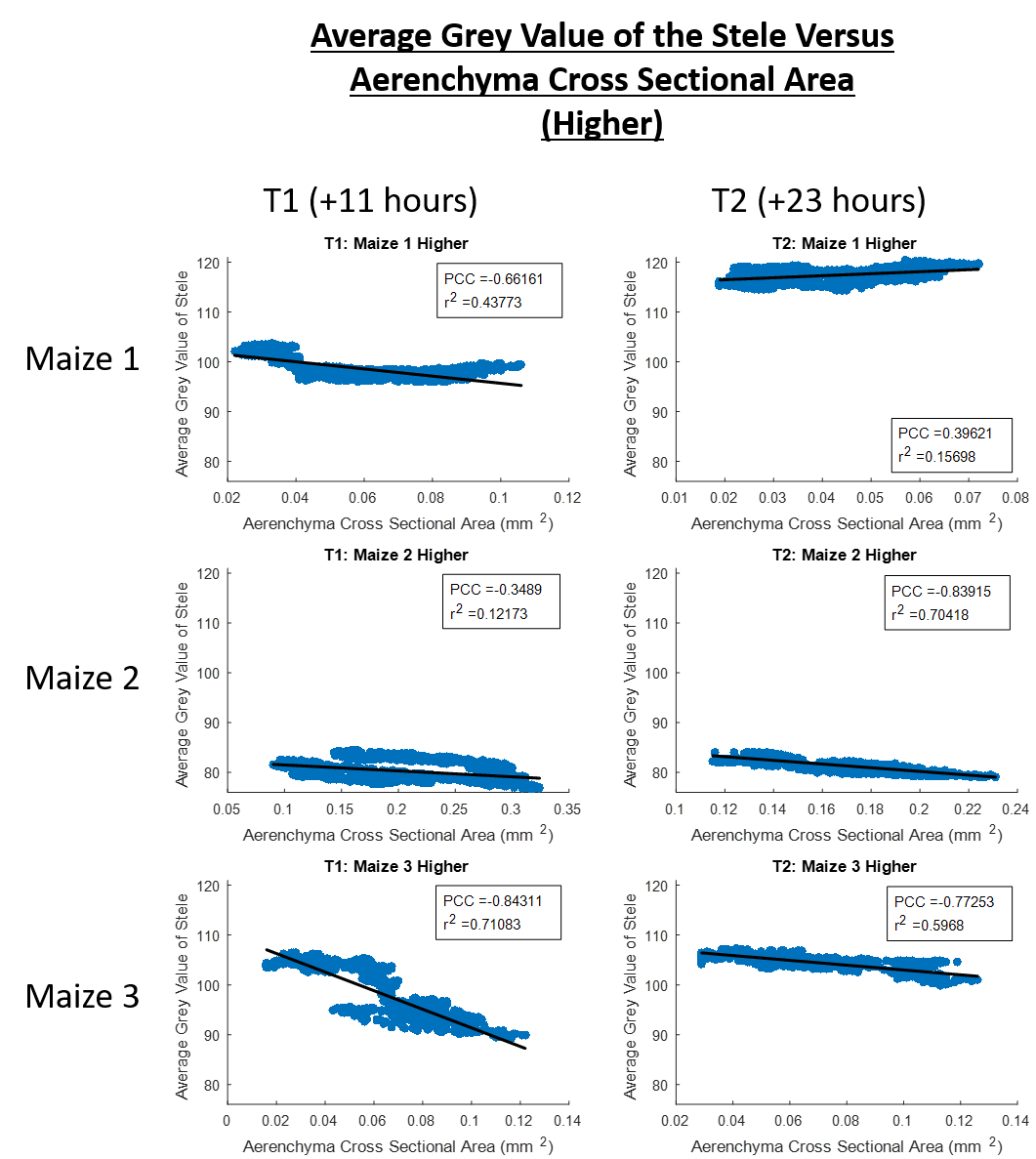
**

**Supplementary Figure S4.** Scatter plots of average grey value of root stele versus aerenchyma cross sectional area over depth for the higher imaged section of roots. Each individual data point on the scatter plots represents the average grey value of the stele and the aerenchyma cross sectional area recorded in a single slice from within the image stack (**Supplementary Figure S1**). T1 is the time point for scans acquired 11 hours after the addition of contrast media and T2 is the time point for scans acquired 23 hours after the addition of contrast media. Maize 1, Maize 2 and Maize 3 are the names of each of the plant samples. The Pearson correlation coefficient, r^2^ value and linear best fit lines are provided for each scatter plot.

**
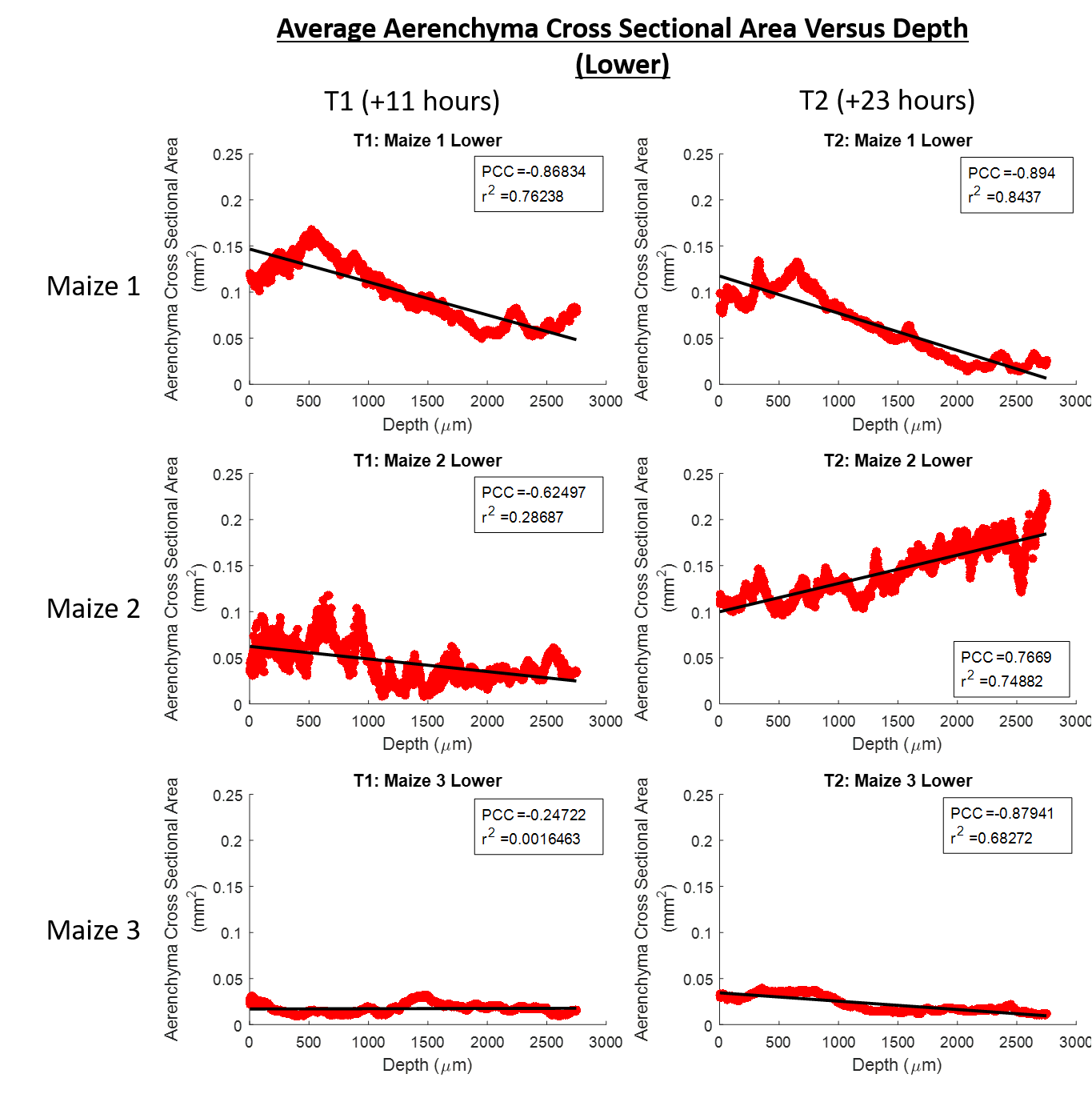
**

**Supplementary Figure S5.** Scatter plots of aerenchyma cross sectional area versus depth down the root growth channel for the lower imaged section of roots. T1 is the time point for scans acquired 11 hours after the addition of contrast media and T2 is the time point for scans acquired 23 hours after the addition of contrast media. Maize 1, Maize 2 and Maize 3 are the names of each of the plant samples. The Pearson correlation coefficient, r^2^ value and linear best fit lines are provided for each scatter plot.

**
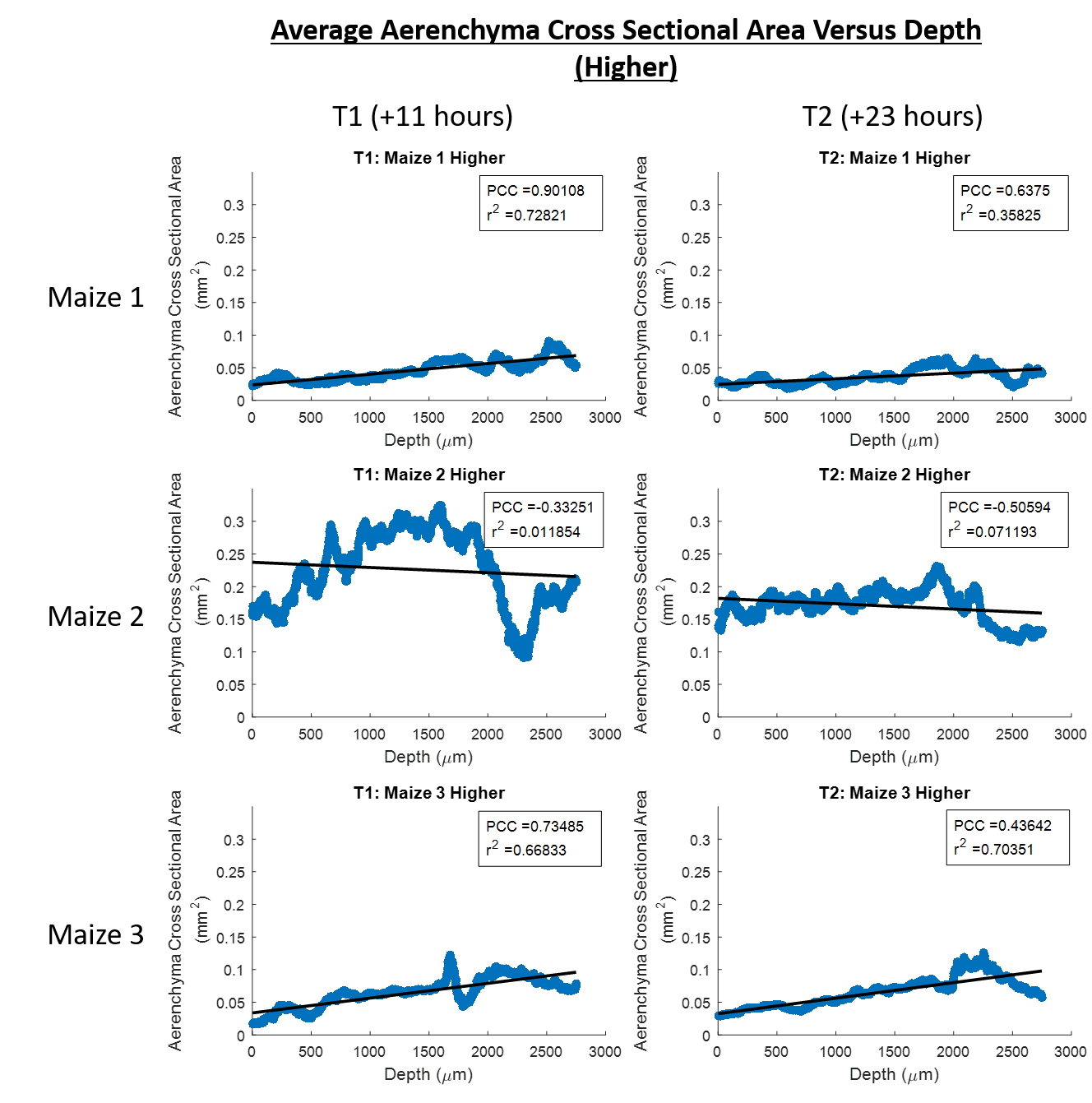
**

**Supplementary Figure S6.** Scatter plots of aerenchyma cross sectional area versus depth down the root growth channel for the higher imaged section of roots. T1 is the time point for scans acquired 11 hours after the addition of contrast media and T2 is the time point for scans acquired 23 hours after the addition of contrast media. Maize 1, Maize 2 and Maize 3 are the names of each of the plant samples. The Pearson correlation coefficient, r^2^ value and linear best fit lines are provided for each scatter plot.


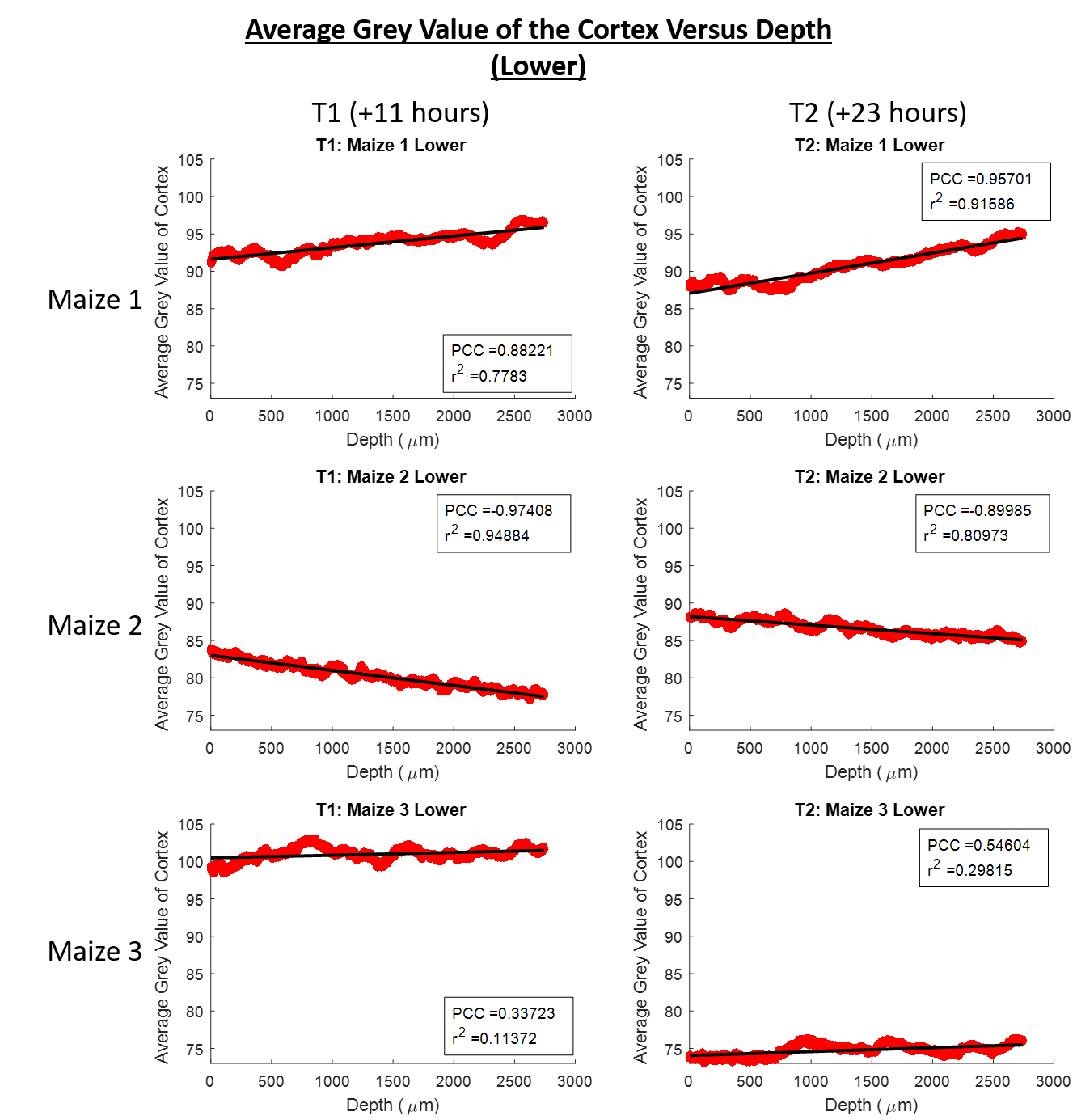


**Supplementary Figure S7.** Scatter plots of average cortex grey value versus depth down the root growth channel for the lower imaged section of roots. T1 is the time point for scans acquired 11 hours after the addition of contrast media and T2 is the time point for scans acquired 23 hours after the addition of contrast media. Maize 1, Maize 2 and Maize 3 are the names of each of the plant samples. The Pearson correlation coefficient, r^2^ value and linear best fit lines are provided for each scatter plot.

**
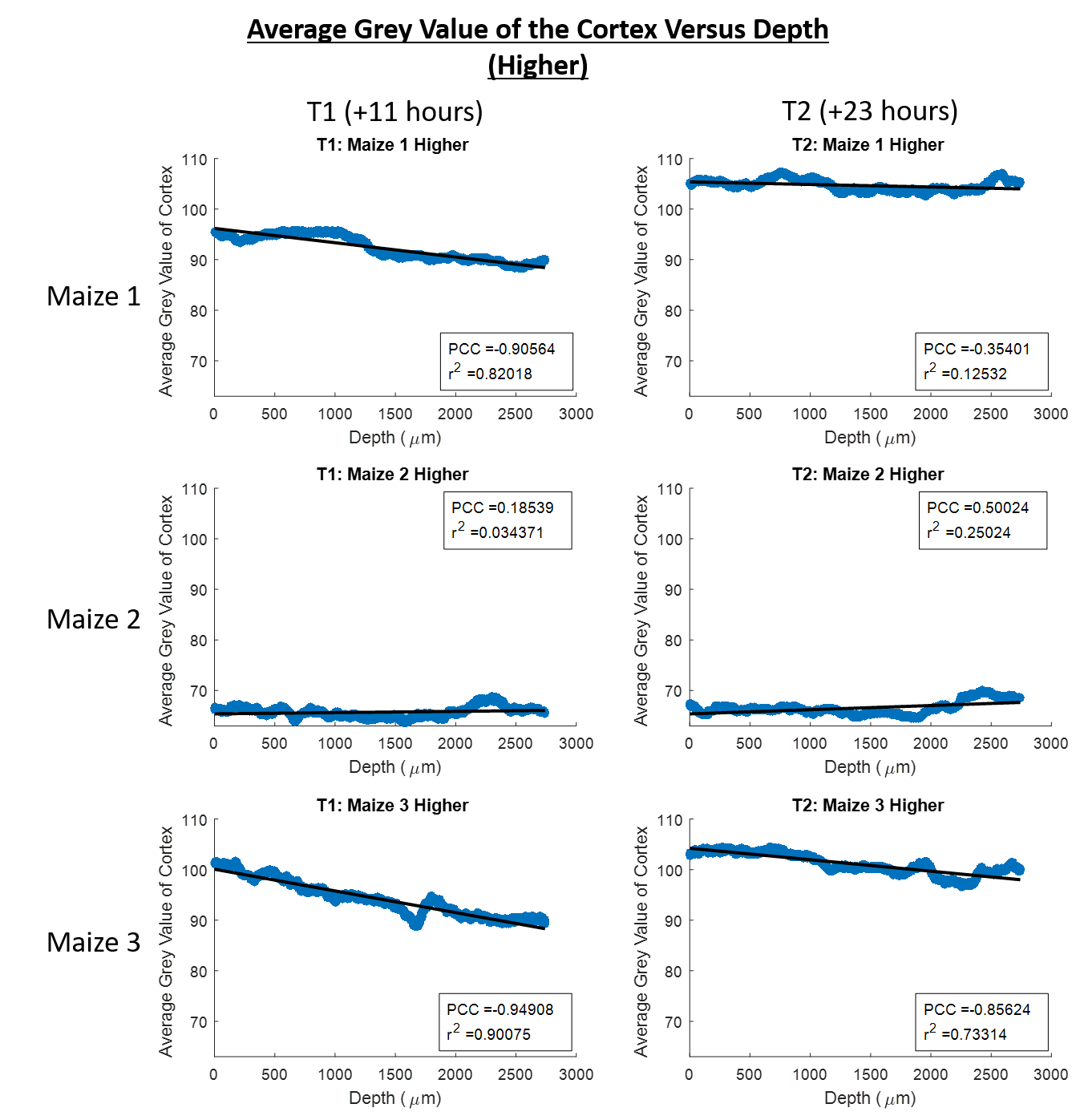
**

**Supplementary Figure S8.** Scatter plots of average cortex grey value versus depth down the root growth channel for the higher imaged section of roots. T1 is the time point for scans acquired 11 hours after the addition of contrast media and T2 is the time point for scans acquired 23 hours after the addition of contrast media. Maize 1, Maize 2 and Maize 3 are the names of each of the plant samples. The Pearson correlation coefficient, r^2^ value and linear best fit lines are provided for each scatter plot.
